# MRI Based Preterm White Matter Injury Classification: The Importance of Sequential Imaging in Determining Severity of Injury

**DOI:** 10.1371/journal.pone.0156245

**Published:** 2016-06-03

**Authors:** Miriam Martinez-Biarge, Floris Groenendaal, Karina J. Kersbergen, Manon J. N. L. Benders, Francesca Foti, Frances M. Cowan, Linda S. de Vries

**Affiliations:** 1 Department of Paediatrics, Imperial College London, London, United Kingdom; 2 Department of Neonatology, Wilhelmina Children’s Hospital, UMCU, Utrecht, the Netherlands; The Research Institute at Nationwide Children's Hospital, UNITED STATES

## Abstract

**Background:**

The evolution of non-hemorrhagic white matter injury (WMI) based on sequential magnetic resonance imaging (MRI) has not been well studied. Our aim was to describe sequential MRI findings in preterm infants with non-hemorrhagic WMI and to develop an MRI classification system for preterm WMI based on these findings.

**Methods:**

Eighty-two preterm infants (gestation ≤35 weeks) were retrospectively included. WMI was diagnosed and classified based on sequential cranial ultrasound (cUS) and confirmed on MRI.

**Results:**

138 MRIs were obtained at three time-points: early (<2 weeks; n = 32), mid (2–6 weeks; n = 30) and term equivalent age (TEA; n = 76). 63 infants (77%) had 2 MRIs during the neonatal period. WMI was non-cystic in 35 and cystic in 47 infants. In infants with cystic-WMI *early MRI* showed extensive restricted diffusion abnormalities, cysts were already present in 3 infants; *mid MRI* showed focal or extensive cysts, without acute diffusion changes. A significant reduction in the size and/or extent of the cysts was observed in 32% of the infants between early/mid and *TEA MRI*. In 4/9 infants previously seen focal cysts were no longer identified at TEA. All infants with cystic WMI showed ≥2 additional findings at TEA: significant reduction in WM volume, mild-moderate irregular ventriculomegaly, several areas of increased signal intensity on T1-weighted-images, abnormal myelination of the PLIC, small thalami.

**Conclusion:**

In infants with extensive WM cysts at 2–6 weeks, cysts may be reduced in number or may even no longer be seen at TEA. A single MRI at TEA, without taking sequential cUS data and pre-TEA MRI findings into account, may underestimate the extent of WMI; based on these results we propose a new MRI classification for preterm non-hemorrhagic WMI.

## Introduction

Cerebral white matter injury (WMI) is the most important form of preterm brain injury, occurring mostly in infants born between 23 and 32 weeks gestation. Before the widespread use of magnetic resonance imaging (MRI) the diagnosis was predominantly made with cranial ultrasound (cUS), and attention mostly drawn to cystic-periventricular leukomalacia (c-PVL) [1]. With increased use of MRI in preterm infants, it has become clear that the spectrum of WMI is much wider and includes more subtle lesions not reliably detected using cUS [2–3].

In some units MRI has become standard of care and is usually performed before discharge or around term equivalent age (TEA) [4]. Other centres are performing 2 MRI scans, the first a few weeks after birth, following clinical stabilization, and the second at discharge or TEA [5]. The MRI scoring systems currently used for assessing WMI are based on the TEA scan and have not been related to earlier cUS or MRI findings. It is likely however that a score based on a scan performed many weeks after the lesion was initiated may result in a different severity score compared to a score based on an MRI obtained soon after the lesion onset. The appearances of WMI change over time, and this change has, so far, been best documented using serial cUS but not by serial MRI [6–9].

Using cUS, localized white matter (WM) cysts, graded as c-PVL grade II, are only seen for a few weeks and often no longer at TEA [6,7]; this may even be the case for infants with extensive grade III c-PVL. Ex-vacuo ventriculomegaly may be seen instead, with irregularly shaped lateral ventricles, at the site of previous cysts.

Whilst the intraventricular hemorrhage (IVH) scoring system of Papile et al. [10] for cUS still holds true to a certain extent using MRI, it is more difficult to make a comparison between the cUS-based PVL classification system and MRI scores currently available for preterm WMI [5,11–12]. The first MRI-based preterm WMI classification by Miller et al. [5] was mainly based on focal signal intensity (SI) changes on T1-weighted imaging, related to the number of punctate high SI lesions, or regions of low SI suggestive of cysts. The more commonly used classification systems of Woodward et al. [11] and the new score by Kidokoro et al. [12] are based on MRI findings around TEA. In the Woodward score white matter cysts were very rare and ventriculomegaly due to post-hemorrhagic ventricular dilatation following a large IVH, or cystic evolution following parenchymal hemorrhage (porencephaly) were part of the WMI score.

The aim of this study was to assess non-hemorrhagic WMI using serial cUS and MRI in a cohort of preterm infants and to propose a new MRI classification system based on these findings.

## Methods

Permission from the medical ethical review committees of the University Medical Center Utrecht (MERC UMC Utrecht) and Hammersmith Hospital–Imperial College London for the current study, and informed parental consent for the MRI were obtained. Patient data were anonymized prior to analysis. Since this was a retrospective study, using MRIs performed as part of standard clinical care, oral consent for the MRI was obtained by the treating physician and any questions or remarks were noted in the charts. No written consent was deemed necessary. The MERC UMC Utrecht waived the need for parental consent for both the use of medical data and for publication of medical images.

### Patients

In a retrospective analysis, we studied preterm infants who were seen in two tertiary neonatal intensive care units (NICU) between December 2003 and December 2014. They were included if they had inhomogeneous echogenicity in the periventricular WM on cUS, seen as non-hemorrhagic punctate white matter lesions (PWMLs) on MRI or when they had c-PVL on serial cUS [6]. Eighty-two infants were identified. This cohort has been partly investigated before for other purposes [2,13]. A subgroup of 35 infants was included in the description of non-cystic, punctate white matter lesions in preterm infants [2]. A second subgroup of 33 infants was studied with DTI and volumetric MRI in order to document acute and remote effects of extensive white matter injury [13].

All infants had 1, and 63 infants (77%) had 2 MRIs in the neonatal period. Infants with WM cysts following a large IVH with an associated parenchymal hemorrhage, (periventricular hemorrhagic infarction) were not eligible for this study.

### Cranial ultrasound

The first cUS was performed as soon as possible after admission to the NICU and repeated 2–3 times during the first week, then once a week until discharge and once again at 40 weeks post-menstrual age. Scanning was performed with an ATL-5000 ultrasound machine (Philips Medical Systems, Best, the Netherlands) with a transducer frequency of 5–8 MHz or a Toshiba Aplio (Toshiba Medical Systems, Zoetermeer, the Netherlands). All infants underwent cUS within 24 hours of the MRI.

### Magnetic Resonance Imaging

Infants diagnosed with inhomogeneous echogenicity in the periventricular WM or c-PVL on cUS were referred for an MRI as soon as they were clinically stable and again, at TEA. MRI was performed on either a 1.5T ACS-NT system or a 3 Tesla whole-body Achieva system (Philips Medical Systems, Best, the Netherlands). Infants were sedated using oral chloral hydrate (30–50 mg/kg). Heart rate, transcutaneous oxygen saturation (Nonin Medical Incorporated, Minneapolis, MN, USA) and respiratory rate were monitored. For hearing protection Minimuffs (Natus Medical Incorporated, San Carlos, CA, USA) and Earmuffs (Em’s 4 Kids, Brisbane, Australia) were used. A neonatologist or physician assistant was present throughout the examination.

On the 1.5T scanner, the routine protocol included conventional axial inversion recovery-weighted and T2-weighted imaging (inversion recovery-weighted TR 4147ms; TI 600ms; TE 30ms; slice thickness 2mm and T2-weighted TR 7656ms; TE 150ms; slice thickness 2mm), as well as diffusion weighted imaging (DWI, single-shot echo planar imaging in 3 orthogonal directions; 25 slices; slice thickness 4mm; TR 3700–5200ms; TE 89ms; b-values of 0 and 1000mm^2^/s, no gap).

On the 3T scanner, the routine protocol included conventional 3D T1 and T2-weighted imaging (at 30 weeks: 3D T1-weighted TR 9.4ms; TE 4.6ms; slice thickness 2mm and T2-weighted TR 8670ms; TE 160ms; slice thickness 2mm; at 40 weeks: 3D T1-weighted TR 9.5ms; TE 4.6ms; slice thickness 1.2-2mm and T2-weighted TR 4847-6293ms; TE 120-150ms; slice thickness 1.2-2mm), as well as DWI (single-shot echo planar imaging in 3 orthogonal directions; 25 slices; slice thickness 3-4mm; TR 2393ms; TE 68ms; b-values of 0 and 800mm^2^/s, no gap).

From June 2009 onwards the susceptibility weighted imaging (SWI) sequence became available (3D gradient-echo sequence with flow compensation, multishot echo-planar imaging; at 1.5T: TR 82ms; TE 40ms, at 3T: TR 52ms; TE 30ms, slice thickness 2mm and EPI factor 3 in both). This sequence was used to distinguish between hemorrhagic and non-hemorrhagic punctate lesions [2].

### Imaging scoring

#### Cranial ultrasound

cUS images were scored using the 4-grade classification by de Vries et al [6].

- Grade I, non-cystic WMI was diagnosed on cUS, as periventricular echogenicity (PVE) present for more than seven days, seen in the coronal and sagittal plane, being as or more echogenic than the choroid plexus. A distinction was made between homogeneous and inhomogeneous echogenicity.
- Grade II, PVE evolving into focal cystic lesions
- Grade III, PVE evolving into extensive cystic lesions
- Grade IV, diffuse echogenicity evolving into periventricular and subcortical cystic lesions

In infants >28 weeks’ gestation, who would not have a routine MRI, the presence of inhomogeneous echogenicity was considered an indication for performing an MRI. Furthermore, all infants who were noted to have cystic evolution on serial cUS had an MRI to confirm the diagnosis and better assess the extent of WMI.

#### MRI

MR images were scored independently by two neonatal neurologists (FC & LdV) each with more than 20 years’ experience in neonatal neuroimaging, blinded to the clinical data and to the cUS results.

At all time-points images were assessed for anatomic development, evidence of IVH, cerebral sinovenous thrombosis (CSVT) or cerebellar injury, abnormal SI in the basal ganglia, thalami and brainstem. The number of PWMLs, their appearance, location and laterality were scored according to Cornette et al [2,14].

In addition, the following abnormalities were documented and scored (S1 Table):

- Early MRI scan (obtained within 2 weeks of birth or the presumed insult): presence, location and extent of DWI abnormalities in the WM, presence of SI changes and cysts on conventional imaging.
- Mid MRI scan (2–6 weeks after birth/presumed insult): presence, number, size, location and side of cystic and non-cystic lesions.
- TEA (39–44 weeks post-menstrual age): presence, number, size and location of cystic lesions, presence/severity of ventriculomegaly, degree of WM loss and reduction in thalamic volume on visual assessment, presence/extent of SI changes suggestive of gliosis; abnormal myelination of the posterior limb of the internal capsule (PLIC). Ventriculomegaly was considered mild when the atrial diameter measured 7.5–10 mm, moderate if the atrial diameter was >10 mm, and severe if the atrial diameter was >14 mm.

### Statistical analysis

Comparison of clinical characteristics between infants with cystic and infants with non-cystic WMI was performed using Mann-Whitney and Fisher’s exact test. Data are shown as mean ± standard deviation, median and range and percentage.

## Results

The main clinical characteristics of the 82 infants are summarized in Table 1.

**Table 1:** Patient characteristics. GA = gestational age, M = male, F = female, SD = standard deviation, * Difference between infants with non-cystic and infants with cystic-white matter injury (WMI).

Characteristics	All infants (n = 82)	Infants with non-cystic-WMI (n = 35)	Infants with cystic-WMI (n = 47)	*P* value*
GA (weeks), median (range)	29.8 (24–35.4)	29.4 (24.7–32.7)	30.0 (24–35.4)	0.037
Sex (M/F)	51/31	16/19	35/12	0.011
Birth weight (g), mean ± SD	1453.3 ± 472.5	1309.1 ± 368.6	1577.1 ± 511.2	0.007
5-minute Apgar score, median (range)	8 (1–10)	8 (1–10)	8 (3–10)	0.6
Blood culture proven sepsis, n (%)	18/78 (23)	10 (29)	8/43 (19)	0.42
Mechanical ventilation >7 d, n (%)	23 (28)	11 (31)	12 (26)	0.62
Patent ductus arteriosus, n (%)	20/78 (26)	13 (37)	7/43 (16)	0.04
Any surgery, n (%)	9/78 (11.5)	4 (11)	5/43 (12)	1.00

### Diagnosis with cranial ultrasound

**Fig 1:**
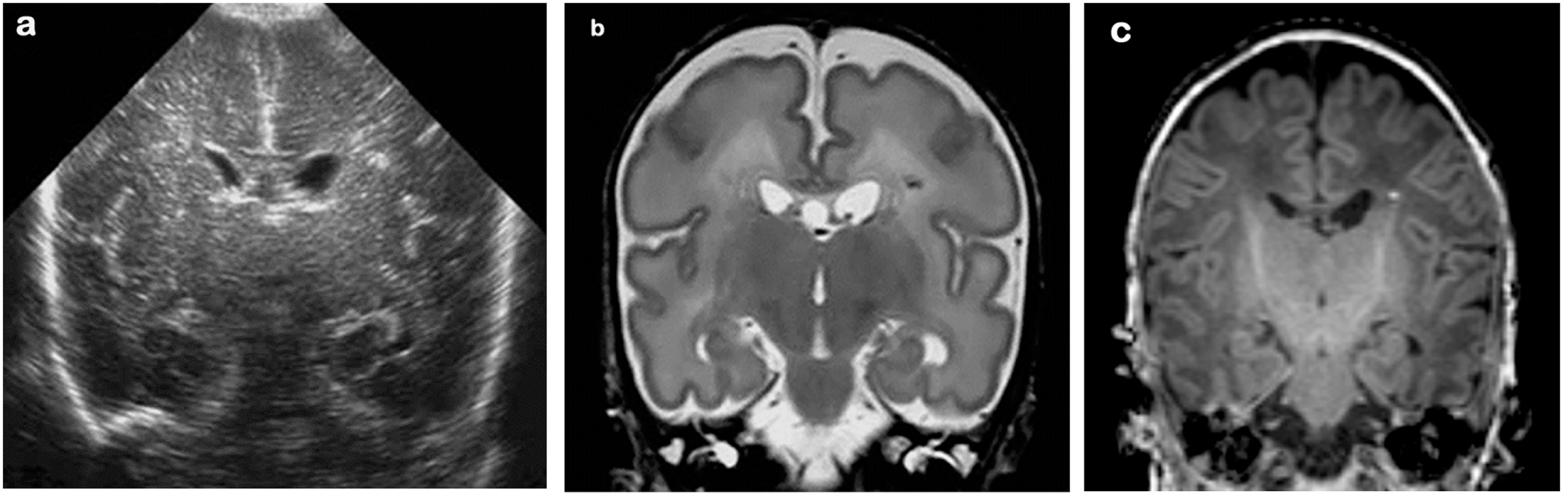
26 week gestation infant; cUS and MRI at 32 weeks post-menstrual age. (a) cUS mid-coronal view, showing a focal lesion in the white matter also seen on early MRI-T2 weighted (b) and at TEA-T1 weighted MRI (c)

Thirty-five infants had inhomogeneous echogenicity in the periventricular WM lasting more than a week, without cystic evolution, and were diagnosed with PVL grade I [6] (Fig 1).

Forty-seven infants developed WM cysts, which were first seen at a median postnatal age of 22 days (range 0–58). Ten of these infants were not initially in our NICUs, but were admitted later following birth abroad (n = 2), referral for necrotising enterocolitis (NEC) surgery (n = 1), with seizures from rotavirus infection (n = 1), for an MRI once the diagnosis was made in a local hospital (n = 6). In 3 infants cysts were present on the first cUS performed on admission; in 34 infants the cysts were noted on their weekly cUS in our 2 units; in 8 infants the diagnosis was made on a repeat cUS at the local hospital following discharge from the level-III NICU, and in 2 during the first follow-up visit at TEA. In 11 infants PVE was first seen beyond the first postnatal week, following a CONS infection (n = 1), viral illness (rotavirus; n = 2), CSVT (n = 1) or NEC (n = 2), or following seizures or pronounced apneas with an onset beyond day 7, without a clear cause (n = 5).

**Fig 2:**
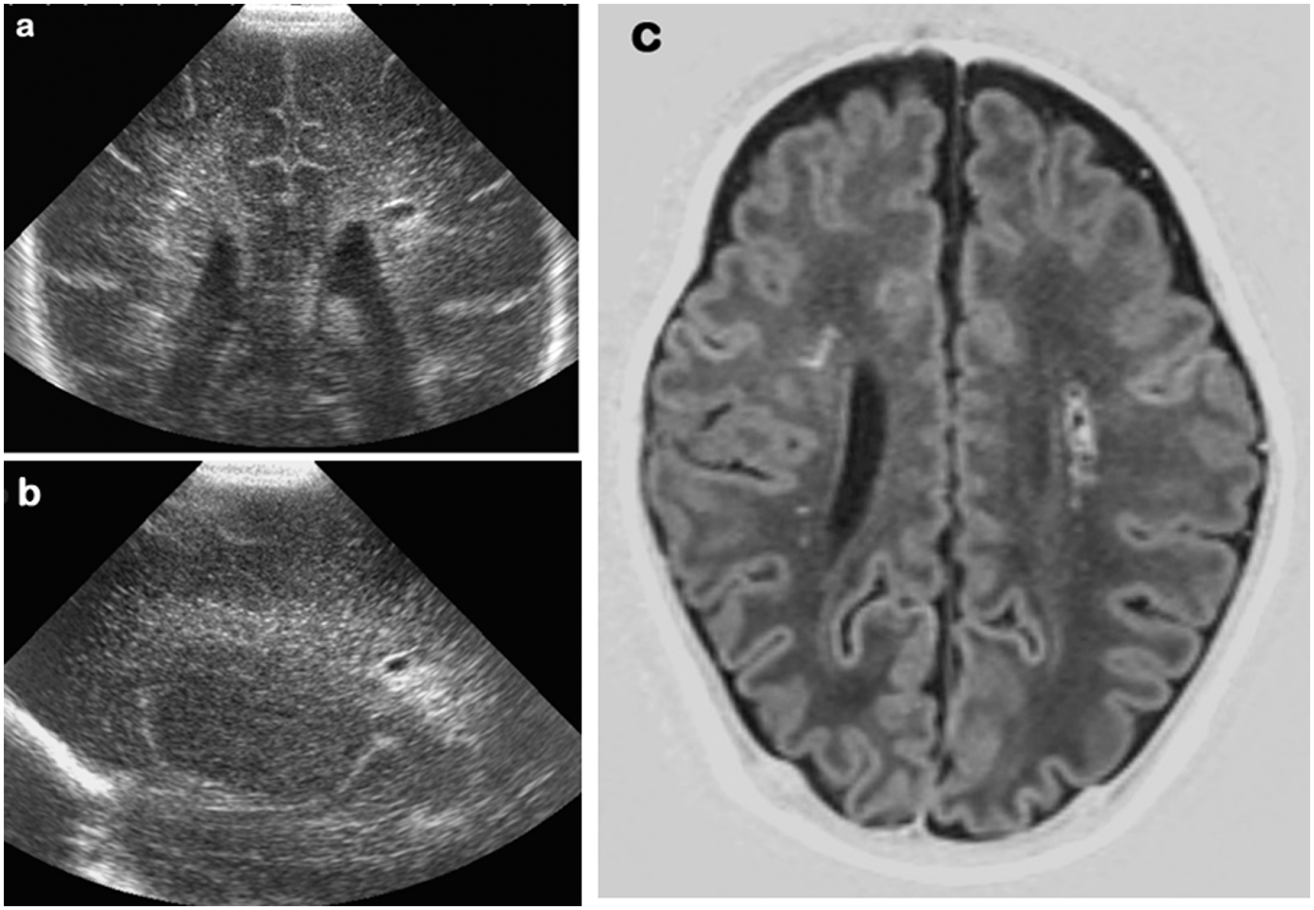
28 week gestation infant; cUS at 5 weeks and TEA-MRI. cUS, coronal (a) and left parasagittal (b) views showing a single cyst and echogenicity surrounding the cyst. T1-weighted MRI at TEA confirming the cyst and several focal lesions of increased signal intensity bilaterally (c).

**Fig 3:**
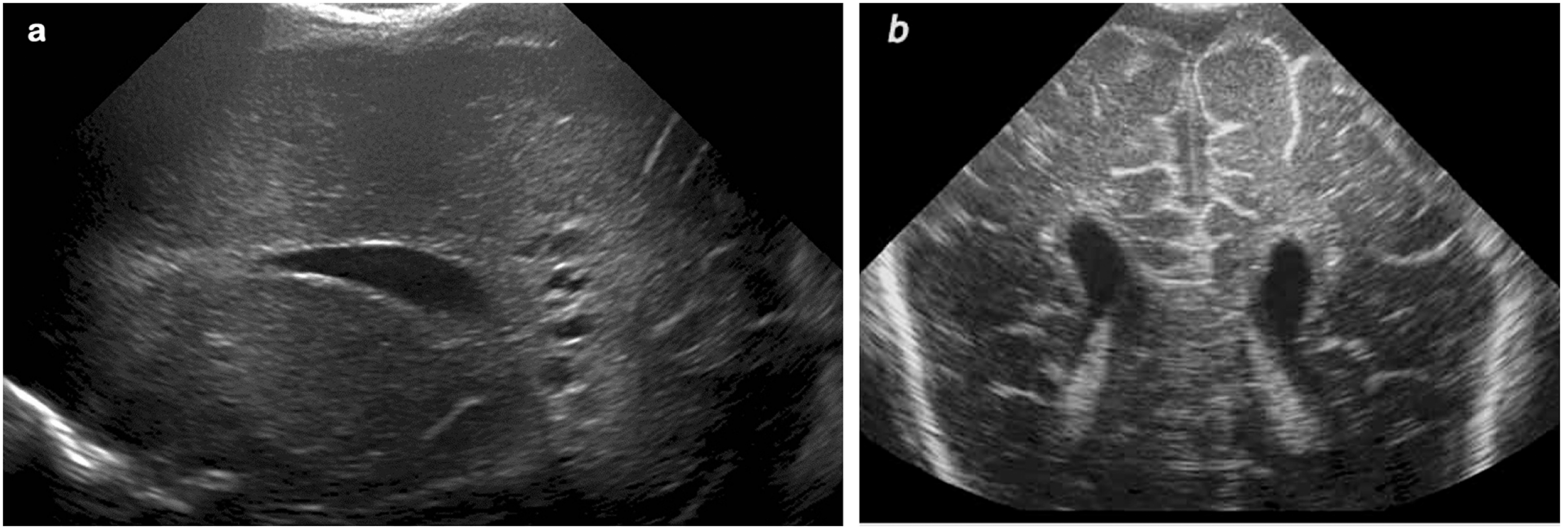
27 week gestation infant. (a) right parasagittal cUS (day 21) showing extensive cysts. The cysts are no longer seen at TEA but there are some punctate lesions of increased echogenicity in the periventricular white matter; there is also white matter loss with sulci abutting the ventricles as well as increased/enlarged extracerebral space (b).

**Fig 4:**
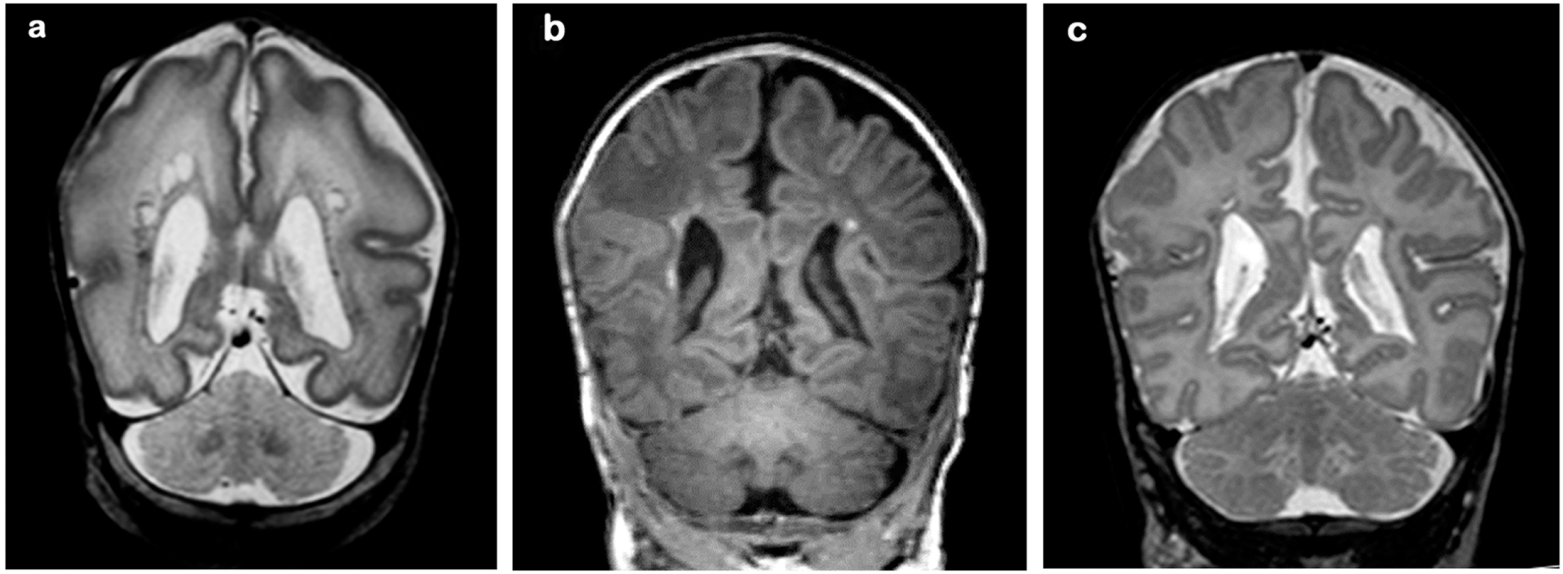
Same infant as above. (a) Early coronal T2-weighted MRI (day 27) showing extensive cysts, more prominent on the right. (b) The cysts are no longer seen on the T1-weighted TEA-MRI but extensive high signal intensity changes and white matter loss are seen instead, more marked on the right side; and (c) a single small cyst on the right is still seen shown on the T2-weighted MRI.

**Fig 5:**
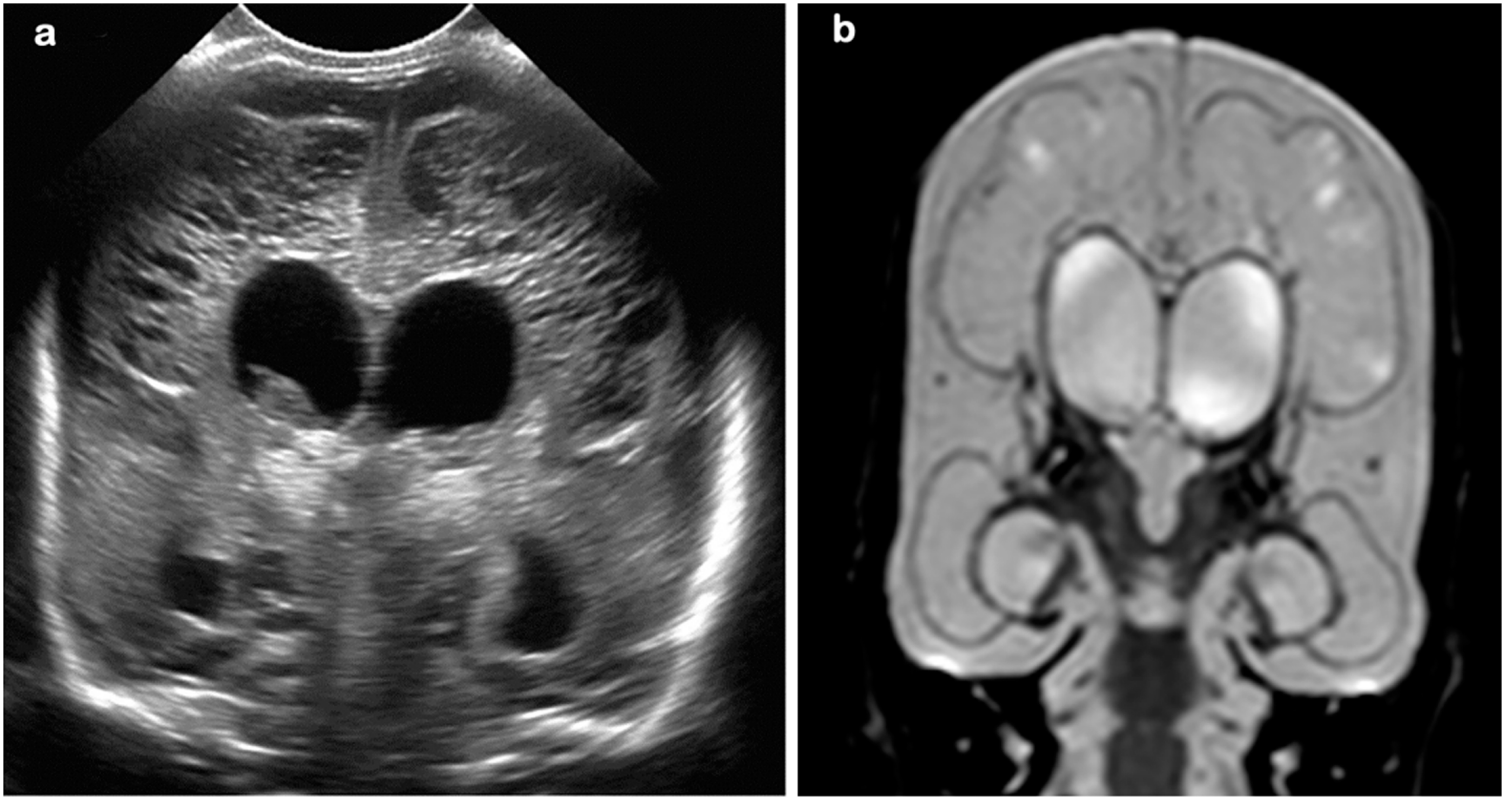
Survivor of monochorionic diamniotic twin pregnancy, who had inhomogeneous echogenicity at birth and showed cystic evolution by day fourteen. Note the discrepancy between the appearance of the cysts on cUS (a) and on the T2-weighted MRI (b), both performed at three weeks. Also note the abnormalities on cUS and MRI in the thalami.

The extent of the cysts was based on the ultrasound classification reported previously [6]. Focal cysts (grade II) were seen in 9 infants, extensive cysts (grade III) in 35 and subcortical cysts (grade IV) in 3 infants (Figs 2–5).

The cysts were bilateral in 40/47 infants, in 3 they were predominantly unilateral, only 4 infants had unilateral cysts (2 focal, 2 extensive).

Cysts were still seen on cUS at TEA in 32/42 (76%) infants with a TEA scan (5 infants died before TEA), but were smaller compared to previous cUS scans in all infants.

### Confirmation with MRI

#### Infants in whom no white matter cysts were detected (PVL grade I; n = 35)

All these infants had 2 MRIs, one either in the first 2 postnatal weeks (early MRI, n = 17) or between weeks 2–6 (mid MRI, n = 18), and all 35 had a scan at TEA. On the early and mid MRI, all infants had PWMLs; these were <6 in 15 infants and ≥6 in 20 infants [2]. Focal diffusion restriction at the site of the PWMLs was seen in 18 infants on the early MRI, but not on the mid MRI. At TEA, PWMLs were no longer seen in 8 infants, 12 infants had <6 and 15 infants had ≥6 PWMLs [2] (Fig 1).

#### Infants with cysts in the white matter (c-PVL grades II-IV; n = 47)

Early MRI: 15 infants had an early MRI; this was obtained at post-mortem during the first week in a set of monozygous twins with an antenatal diagnosis of multicystic encephalomalacia. The MRI was performed within two weeks of birth in another 8 infants; and within 2 weeks of an insult occurring later in the neonatal period (CSVT, NEC; CONS, rotavirus) in 5. In all infants except for the twins MRI was performed because of inhomogeneous echogenicity on cUS; all 13 infants showed extensive confluent DWI-MRI changes with subsequent cystic evolution. In 3 infants a combination of restricted diffusion as well as cystic lesions was seen.

Mid-MRI: 12 infants had an MRI 2–6 weeks after birth. All but two had an insult apparently around the time of birth. Extensive cysts were seen with cUS and confirmed in all with MRI. Restricted diffusion changes were no longer present. In one infant the cUS underestimated the extent of the cysts seen on MRI.

**Fig 6:**
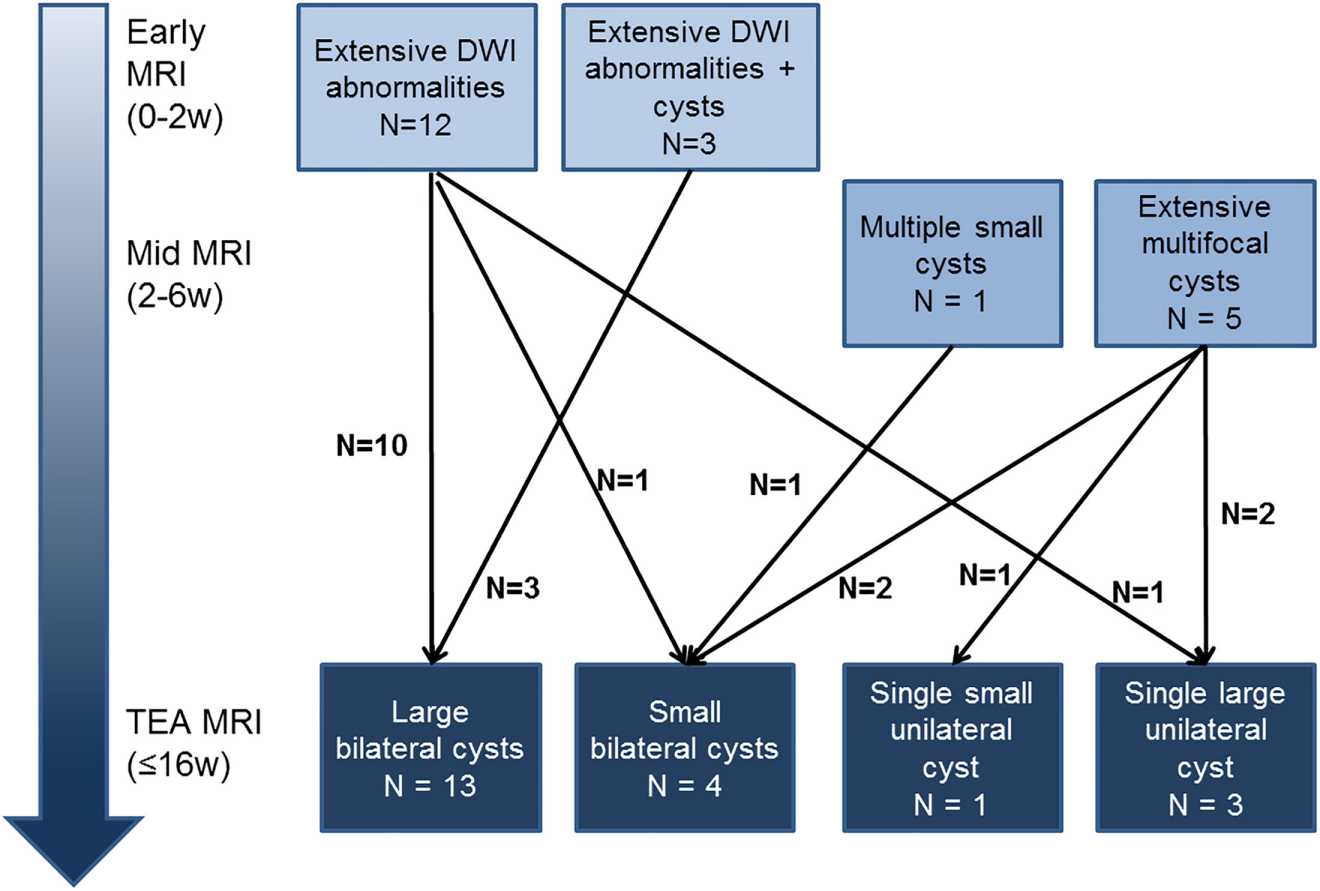
Diagram showing evolution of cystic lesions between early/mid MRI and TEA MRI in the 22 infants with early extensive cysts and at least 2 MRIs in the neonatal period.

TEA-MRI: 42 infants had a TEA-MRI. Five infants (the 3 with extensive subcortical cysts, 1 who had an early MRI and 1 who had a mid-MRI) died before TEA following a decision to redirect care. Extensive periventricular cysts were present in 22 infants; they were bilateral in 21 and unilateral in 1, who previously had had predominantly unilateral DWI abnormalities). Three infants had a large unilateral single cyst, no longer seen with cUS in 1 and had focal small cysts, also recognized on cUS as focal cysts (Fig 2). Five infants had small cysts (unilateral in 1), in spite of extensive bilateral cysts seen on mid-MRI and/or cUS. In 4 infants, who had focal cysts on earlier cUS, cysts were no longer seen on the TEA cUS and/or MRI. Of the 22 infants with 2 MRI (early/mid + TEA), cysts were significantly smaller and/or less extensive at TEA in 7 (32%). (Fig 6)

#### Associated MRI findings in infants with cysts

*Ventriculomegaly* was present in 35 infants; it was measured at the atrium and mild (7.5-10mm) in 24, moderate (>10mm) in 9 and severe (>14mm) in 2. The ventricles were irregularly shaped, especially in infants whose cysts were less obvious or no longer seen. No ventriculomegaly was seen in 7 infants.

*White matter loss*, with sulci abutting the ventricles, was seen in all 42 infants who had a TEA-MRI.

**Fig 7:**
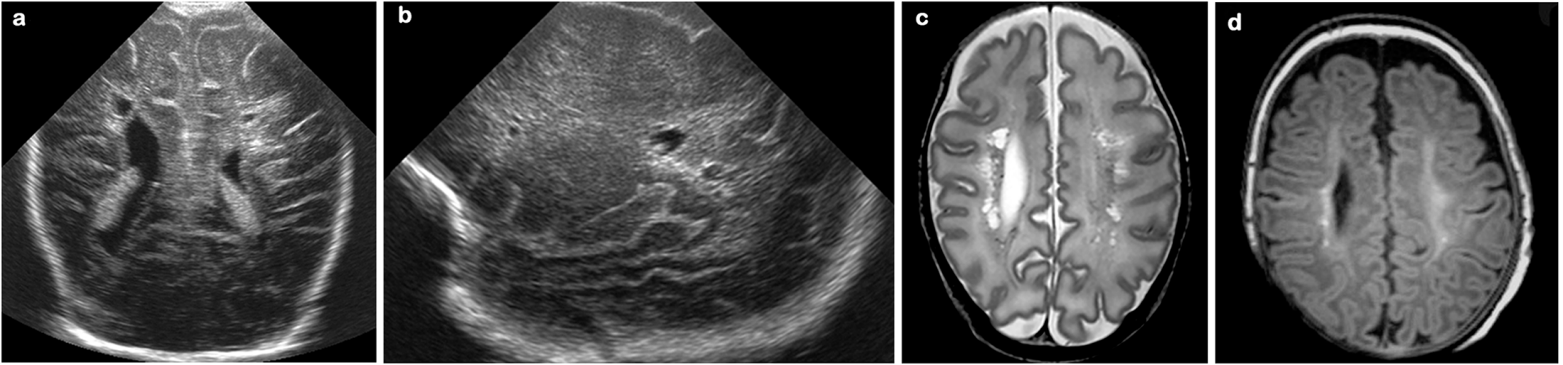
31 week gestation infant; cUS performed 4 weeks after birth. Coronal (a) and right parasagittal view (b) showing extensive cysts, more so on the right. (c) First T2-weighted MRI performed 4 weeks after birth showing extensive cysts, more prominent on the right. (d) The cysts were no longer seen on the T1-weighted TEA-MRI; extensive high SI changes and white matter loss are seen instead, also more marked on the right side.

*Increased SI in the WM* suggestive of periventricular gliosis was present in 38 infants, being focal in 34 and extensive in 4 infants. In all infants these changes were seen bilaterally, although clearly asymmetrical in 5 (Figs 4 and 7).

**Fig 8:**
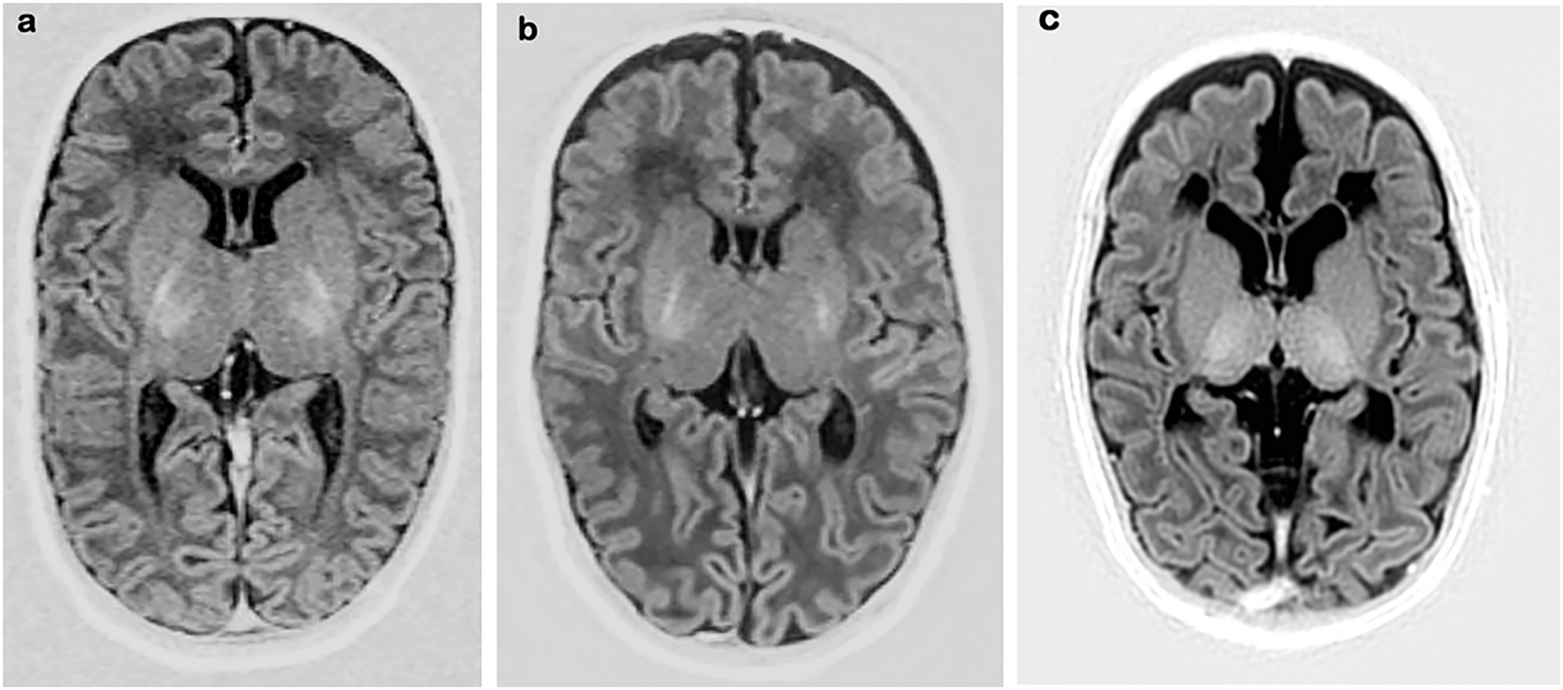
Inversion Recovery images, performed in three preterm infants at TEA. (a) Normal myelination of the PLIC. (b) Sparse myelination, slightly better on the right; and (c) no myelination.

*Myelin in the PLIC* appeared normal in 3 infants. Myelin was present although not entirely adequate for post-menstrual age in 6 infants. Myelin was present but sparse in 5 infants (asymmetric in 2). The SI from myelin in the PLIC was clearly abnormal or absent in 25 infants (Fig 8).

*Other pathologies*: IVH was seen in 8 infants (2 with c-PVL-II, 6 with c-PVL-III). IVH was grade I in 2 infants, grade II in 5 and grade III in 1. Punctate cerebellar hemorrhages were present in 3 infants, all of whom had c-PVL-III. Seven infants had abnormal SI in the basal ganglia-thalami on their early or mid MRI (3 infants with c-PVL-IV and 4 infants with c-PVL-III), and 1 had bilateral abnormal SI in the thalami at TEA. Reduction in thalamic volume on visual assessment was present in 32 infants. One infant with c-PVL-III showed signs of CSVT when scanned 22 days after birth.

## Discussion

We have for the first time described sequential cUS combined with serial MRI findings in preterm infants with well-documented non-hemorrhagic WMI, previously referred to as PVL [3,6]. We have shown that MRI changes depend on the period elapsed between the presumed insult and the MRI and that in a significant proportion of infants the number and size of cystic lesions is reduced at TEA compared to previous scans. Our results strongly suggest that a single MRI at TEA in preterm infants with WMI may underestimate the severity of the injury. Our data show that in order to optimally interpret an MRI scan performed at TEA information about presumed time of insult as well as severity of preceding lesions is required.

Based on these findings, we propose a new classification system for preterm non-hemorrhagic WMI, taking into account the timing of the MRI in relation to the time of the presumed insult (Table 2). The classification system is based on 82 infants, admitted to 2 tertiary NICUs during the last decade, and can be used in the acute phase within the first 2 weeks after the presumed insult, in the intermediate stage (2–6 weeks) and at TEA (or up to 16 weeks after the presumed insult). Within 2 weeks cysts are unlikely to be seen, focal or extensive DWI changes may be present; between 2–6 weeks cysts will have developed in infants with moderate or severe WMI and ventriculomegaly may have started to become apparent, DWI changes are no longer seen; at TEA cysts may still be there or may already have been replaced by early gliosis, white matter loss and ventriculomegaly. Whether cysts will still be present or not at TEA will depend not only on the severity of the WMI, but on the number of weeks since the initial insult.

**Table 2:** MRI classification for preterm white matter injury. BGT: Basal-ganglia thalami, DWI: Diffusion weighed imaging, PLIC: Posterior limb of the internal capsule, PWMLs: Periventricular white matter lesions, SI: Signal intensity, TEA: Term equivalent age, VM: Ventriculomegaly.

	First 2 wks after birth/insult	2–6 wks after birth/insult	TEA
**Grade Ia**	<6 focal PWMLs and focal DWI abnormalities	<6 PWMLs	PWML<6. Focal increased SI of the WM on T1. Symmetrical age appropriate or nearly age appropriate myelination of the PLIC
**Grade Ib**	≥6 focal PWMLs and focal DWI abnormalities	≥6 PWMLs	≥6 PWMLs. Same as above
**Grade II**	≥6 PWMLs; focal and some confluent DWI abnormalities	≥6 PWMLs / focal periventricular cysts	Focal periventricular cysts and/or at least 2 of the following: mild VM measured at the atrium (7.5-10mm); irregularly shaped ventricles; few focal increased SI WM lesions on T1; (sparse) myelination of the PLIC
**Grade III**	Extensive (confluent) DWI abnormalities	Extensive periventricular cysts	Extensive periventricular cysts, and/or at least 2 of the following: decreased WM volume and mild-moderate VM (>10mm); irregularly shaped ventricles; extensive increased SI WM lesions on T1; sparse or no myelination of the PLIC
**Grade IV**	Extensive (confluent) DWI abnormalities extending into the subcortical white matter	Extensive periventricular and subcortical cysts	Extensive periventricular and subcortical cysts, and/or at least 2 of the following: moderate-severe VM (>14mm); severe complete loss of WM; BGT involvement; extensive increased SI WM lesions on T1; no myelination of the PLIC

We have not included infants with a parenchymal hemorrhage, in contrast to previous classification systems [11,12], where ventricular dilatation following a large IVH or cystic evolution following parenchymal hemorrhage (porencephaly) were also included in the WMI score.

We did not include diffuse excessive high signal intensity (DEHSI) of the white matter in the classification system as this finding is only seen at TEA, and we were performing a longitudinal study of infants with WMI, usually diagnosed in the early neonatal period. Although DEHSI might be a correlate of early echogenicity in the periventricular WM, data on the association between DEHSI and later outcome are inconsistent [15,16]. To understand the real correlation between DEHSI and early WM changes, it would be necessary to MR scan routinely all preterm infants in the first few weeks after birth and then at TEA, as DEHSI has been described in up to 90% of preterm infants [16].

We have shown that preterm WMI assessed from MRI appearances changes over time in a way not dissimilar to that described with cUS. The presence of early confluent DWI changes preceded the development of cysts in all infants who had early scans and no cysts were seen to develop when early DW changes were only focal and limited to the site of the punctate lesions. Inder et al were the first to document preterm DWI WM abnormalities preceding definite injury on conventional MRI and cUS [17]. Since then, others have confirmed the sensitivity of early DWI changes for detecting preterm cystic-WMI [18,19]. Our data strongly suggests that in infants with inhomogeneous PVEs an early MRI with DWI will show the full extent of the injury and will help in the prediction of cystic evolution

In about one third of the infants, the diagnosis of cystic-WMI was made after discharge to the local hospital, stressing the importance of continuing cUS examinations until discharge home [4–8]. In one fourth of infants the presumed onset of WMI was beyond the first week after birth, related to NEC, sepsis, viral illness, or CSVT [20, 21]. A significant reduction in the size and/or extent of the cysts was seen in a considerable proportion of infants at TEA compared to earlier scans and in 4 infants cysts were no longer seen at TEA. Irregularly shaped ex-vacuo ventriculomegaly with greater dilatation depending on the site of the previous cysts was seen instead in most cases. Focal or more extensive high SI areas, suggestive of early gliosis, were also very common at TEA. Reduced thalamic volumes were observed in two thirds of infants at TEA; this visual assessment correlated with volumetric analyses in a subgroup of the same cohort showing significantly reduced thalamic volumes in infants with cystic-WMI compared to controls [13].

Although the incidence of cystic-WMI, diagnosed with cUS, has decreased, [22–24] this remains the most important type of preterm brain injury leading to cerebral palsy and other neurodevelopmental impairments; thus a precise diagnosis neonatally is essential in order to provide adequate counselling and follow-up care. Serial cUS is very useful for showing the evolution of lesions and therefore important in establishing the onset and maximum extent of injury. While there are no major differences in scoring the severity of preterm hemorrhage between cUS and MRI, our data show that the differences between serial cUS findings and TEA MRI are more apparent with more important diagnostic implications for non-hemorrhagic cystic-WMI.

This study has several limitations: it is not prospective and while all infants with a gestational age <28 weeks had a routine MRI at TEA, and since 2008 also at 30–31 weeks post-menstrual age if they were clinically stable, infants >28 weeks’ gestation had only an MRI when serial cUS examinations were suggestive of WMI. Only those infants in whom MRI confirmed WMI were eligible for this study. However, as our cUS protocol includes many examinations starting soon after birth and until TEA, it is unlikely that significant WMI has been missed. This is not a population or a hospital-based study; the number of infants and the distribution of WMI severity in this study do not reflect the incidence and magnitude of this problem in preterm infants in clinical practice; this cohort should be seen instead as the largest sample so far available of a comprehensive sample of infants with non-hemorrhagic WMI. Another limitation is that we could only include a relatively small number of infants with focal cystic-WMI and multicystic WMI so the MRI changes in these subgroups are not as well characterized as for infants with extensive cystic-WMI.

The main strength of this study is the large number of cUS and MRI examinations, performed over an extended period in infants with different degrees of well-characterized, non-hemorrhagic WMI. This allowed us to describe the natural evolution of this type of injury throughout the neonatal period and to attain a better understanding of the nature and significance of the abnormalities that are commonly observed on MRI in preterm infants at TEA. These observations have made us aware that the absence of cysts at TEA does not exclude the presence of severe WMI. Other findings, such as ventriculomegaly, reduced thalamic volume and early gliosis may be seen instead and will aid in making the appropriate diagnosis. We are now analysing the relationship between this scoring system, follow up MRI scans and neurodevelopmental outcomes.

In **conclusion,** our results emphasize the need for sequential cUS examinations in preterm infants until TEA. MRI can be a very useful auxiliary tool in this population, but in order to be correctly interpreted, MRI findings should be scored taking into account not only the presence of cysts at TEA, but the presumed time of injury, the severity of the preceding lesions seen with cUS and MRI and the presence of other signs indicative of previous injury. The scoring system we propose can be used for a more accurate assessment of the severity of preterm WMI.

## Supporting Information

S1 Table MRI Collection Form. (DOCX)

S1 Dataset Clinical and imaging data. (XLSX)
