# Supplementary material for: MRI Based Preterm White Matter Injury Classification: The Importance of Sequential Imaging in Determining Severity of Injury
Source: PLoS One. 2016 Jun 3;11(6):e0156245. doi: 10.1371/journal.pone.0156245 (PMC4892507; doi:10.1371/journal.pone.0156245)
Supplement: S1 Table — (DOCX) [file pone.0156245.s001.docx]

| **Table 1. MRI Data collection form** | | | | | | |
| --- | --- | --- | --- | --- | --- | --- |
| ID number: | |  | | | | |
| DOB: | |  | | | | |
| Gestational age: | |  | | | | |
| **Early & mid MRI** | Postnatal age | | |  | | |
|  | Postmenstrual age | | |  | | |
|  |  | | | | | |
|  | DWI abnormalities WM | | No | |  | |
|  |  |  | Yes | | Unilateral |  |
|  |  |  |  |  | Bilateral |  |
|  |  |  |  |  | Focal / punctate |  |
|  |  |  |  |  | Extensive |  |
|  | DWI abnormalities PLIC | | No | |  | |
|  |  |  | Yes | | Unilateral |  |
|  |  |  |  | | Bilateral |  |
|  | DWI abnormalities peduncle | | No | |  | |
|  |  |  | Yes | | Unilateral |  |
|  |  |  |  |  | Bilateral |  |
|  | Germinal matrix haemorrhage | | No | |  | |
|  |  |  | Yes | | Comments |  |
|  | Intraventricular haemorrhage | | No | |  |  |
|  |  |  | Yes | | Grade II |  |
|  |  |  |  |  | Grade III |  |
|  | Cerebellar lesions | | No | |  | |
|  |  |  | Yes | | Punctate |  |
|  |  |  |  |  | Large |  |
|  |  |  |  |  | Vermis involvement |  |
|  |  |  |  |  | Unilateral |  |
|  |  |  |  |  | Bilateral |  |
|  | WM cysts | | No | |  | |
|  |  |  | Yes | | Unilateral |  |
|  |  |  |  |  | Bilateral |  |
|  |  |  |  |  | Focal |  |
|  |  |  |  |  | Extensive |  |
|  |  |  |  |  | Comments |  |
| Comments: | | | | | | |
| **TEA MRI** | Postnatal age | | |  | | |
|  | Postmenstrual age | | |  | | |
|  |  | | | | | |
|  | WM cysts | | No | |  | |
|  |  |  | Yes | | Focal | Small |
|  |  |  |  |  |  | Large |
|  |  |  |  |  | Extensive |  |
|  |  |  |  |  | Unilateral |  |
|  |  |  |  |  | Bilateral |  |
|  |  |  |  |  | Site | Frontal |
|  |  |  |  |  |  | Parietal |
|  |  |  |  |  |  | Occipital |
|  |  |  |  |  |  | Temporal |
|  | Ventriculomegaly | | No | |  | |
|  |  |  | Yes | | Ventricular width, right |  |
|  |  |  |  |  | Ventricular width, left |  |
|  |  |  |  |  | Unilateral |  |
|  |  |  |  |  | Bilateral |  |
|  | White matter loss | | No | |  | |
|  |  |  | Yes | | Unilateral |  |
|  |  |  |  |  | Bilateral |  |
|  |  |  |  |  | Severity |  |
|  | Increased signal intensity on T1 (suggestive of gliosis) | | No | |  | |
|  |  |  | Yes | | Focal |  |
|  |  |  |  |  | Extensive |  |
|  |  |  |  |  | Unilateral |  |
|  |  |  |  |  | Bilateral |  |
|  | PLIC - Right | | Normal | |  | |
|  |  | | Abnormal | | Sparse |  |
|  |  | |  |  | Absent |  |
|  |  | |  |  | Abnormal shape /SI |  |
|  | PLIC- Left | | Normal | |  | |
|  |  |  | Abnormal | | Sparse |  |
|  |  |  |  |  | Absent |  |
|  |  |  |  |  | Abnormal shape /SI |  |
|  | Thalamic volume | | Right | | Normal | Decreased |
|  |  |  | Left | | Normal | Decreased |
|  | Other abnormalities  Comments | | | | | |
